# Deformation Modeling and Simulation of a Novel Bionic Software Robotics Gripping Terminal Driven by Negative Pressure Based on Classical Differential Algorithm

**DOI:** 10.1155/2022/2207906

**Published:** 2022-05-06

**Authors:** Yinuo Chen, Ligang Yao, Zhenya Wang

**Affiliations:** School of Mechanical Engineering and Automation, Fuzhou University, Fuzhou 350108, China

## Abstract

A general pneumatic soft gripper is proposed in this paper. Combined with the torque balance theory, the mathematical theoretical model of bending deformation of soft gripper is established based on Yeoh constitutive model and classical differential geometry. Assuming that the pressure in each inner cavity is evenly distributed, the input gas is in an ideal state, which is approximately treated as an isothermal condition, and all orifices experience blocked flow. In addition, compared with the mechanical work of gas, the energy related to gas flow and heat transfer is negligible. The nonlinear mechanical properties of silicone rubber are studied. It is regarded as isotropic and incompressible material, which is characterized by strain energy per unit volume. The material constant coefficients *C*_10_ and *C*_20_ are determined through the uniaxial tensile test, and the software gripper is simulated on the ABAQUS platform. The bending deformation models of grippers with three different force-bearing cavity structures are analyzed and compared, and the software clamping structure with the bending deformation most in line with the application conditions is selected. The limit input air pressure of the gripper and the situation of enveloping the clamping target object are analyzed. Through the bending deformation experiment, the maximum deformation angle is 72.4°. The relative error between the simulation analysis data and the prediction results of the mathematical model is no more than 3.5%, which verifies the effectiveness of the simulation and the correctness of the mathematical theoretical model of bending deformation. The soft manipulator proposed in this paper has good adaptability to grasping objects of different shapes and sizes. The minimum diameter of the target object that can be clamped is 0.1 mm. It can clamp the object weighing up to 1 kg. It has compact size, light weight, high ductility, and flexibility.

## 1. Introduction

Traditional rigid robots have the characteristics of high precision, fast response, and high strength. However, due to the poor ductility and flexibility of traditional rigid robots, there are certain safety hazards in human-machine interaction [1], and there are also great limitations in versatility. In recent years, soft robots made of soft materials such as silicone rubber have received wide attention due to their excellent environmental adaptability, safety, and versatility [2–4]. As an important terminal for soft robots to interact with the outside world, flexible manipulators have triggered a lot of research at present [5, 6]. For manipulators, soft materials with good deformation allow them to deform appropriately to fit the surface of the target object [7–10]. Different types of general-purpose soft grippers have been developed in recent years, such as fluid-driven (pneumatic [11–14] or hydraulic [15–17]) elastic grippers, vacuum particle interference grippers [18, 19], electroactive material grippers [20–22], and cable-driven grippers [23–25]. Different from the traditional rigid structure, how to drive the soft structure reasonably to achieve the desired motion pattern has always been a difficult point in the design of soft manipulators. The current driving methods mainly include physical driving and chemical driving. The chemical drive is mainly driven by chemical fuel [26], and the physical drive mainly includes pneumatic drive [26, 27] and cable drive [28–30]. Among them, most of the pneumatic drives use air, which has the advantages of light weight, low price, and no pollution to the environment, so they are favored by researchers. At the same time, due to the flexibility of the material, how to calculate the force exerted on the structure and control the grasping accuracy of the soft manipulator has also become one of the research focuses. At present, the open-loop control or feedback control is often used in the control and optimization design of software manipulator. There is often some unknown dynamic information in the model of software manipulator, so it is necessary to estimate the uncertainty, and the neural network [31] can approximate any nonlinear function, which makes it an effective method for adaptive unmodeled dynamics. In addition, the neural network can also be combined with the backstepping method and applied to the control problems of various high-order complex systems [32]. Reference [33] proposed a new adaptive control to deal with the uncertainty of system parameters. Reference [34] uses camera calibration technology and target recognition technology, uses the PCC inverse dynamics model and image Jacobian matrix to deduce the driving force configuration of the driving space, and realizes the visual servo control of the software manipulator. It is seen that the video conversion in standard Phase Alternating Line (PAL) 576i format to standard video of Video Graphics Array (VGA)/Super Video Graphics Array (SVGA) [35] can process images efficiently under the condition of low resource utilization, meet the flexible real-time video processing, and facilitate the control of the software arm.

Flexible Fluid Actuator (FFA) is a new type of flexible and adaptive soft actuator [36]. There are two main topologies for this fluid-driven elastomer gripper: a biomimetic finger-like soft-body gripper and a granular variable-stiffness gripper. The bionic finger-like soft gripper mainly uses low stiffness to wrap the adaptive object and mostly grasps the target object through frictional force [37]. This type of gripper is limited by the normal force or bending stiffness during bending. These characteristics usually depend on internal fluid pressure and are coupled with each other [38, 39]. Shepherd et al. [40] proposed a pneumatic soft actuator. A series of forced air chambers were set up inside the actuator, and the pressure was input to the chamber to expand the force, thus driving the soft actuator to produce bending deformation. This kind of driving mode has the characteristics of light weight, fast response speed, high safety, good speed characteristics, and energy density, but there are also some shortcomings. The increase of air pressure leads to the change of air chamber volume, which will produce an irregular “bubbling” phenomenon without adding a limiting layer, which will affect the movement of the manipulator in an unstructured environment. Secondly, the change of air chamber volume leads to the nonlinear change of air pressure with time, which increases the difficulty of real-time and accurate control of the manipulator. Marchese et al. [41] used multiple pneumatic artificial muscles (PAM) in series and parallel to simulate the movement of the human wrist joint, proposed two structural design schemes, and analyzed and compared their performance. Pneumatic artificial muscle has the advantages of small volume, large power/weight ratio, and low cost. It can provide large output torque, but there are also some problems. The movement form is single, and the pneumatic artificial muscle cannot actively realize the bending movement, which needs the cooperation of multiple pneumatic artificial muscles. The resolution is low, and the modeling is difficult. Due to the friction between the elastic inner tube and the fiber of the pneumatic artificial muscle and the inelastic deformation of the elastic inner tube, the McKibben pneumatic muscle will have a certain hysteresis and threshold air pressure. Ilievski et al. [42] designed a software gripper with six silicone soft claws by using the expansion characteristics of a pneumatic grid. It has high versatility and flexibility and can grab and loosen objects by inputting positive pressure and negative pressure. At present, Soft Robotics has pushed the software gripper to the market. Brown et al. [18] designed a universal semiactive actuator fixture based on a blocking mechanism, which can use particle blocking to grab objects with irregular outer surfaces. The advantage of this passive blocking method is that it can realize the variable-stiffness function of software module without an additional negative pressure device, but the stiffness cannot be changed at will, which limits the operation ability of the software manipulator. Variable-stiffness grippers have higher strength but are less versatile for the shape of the target object, and the gripping efficiency may be reduced when the outer surface of the target object is rough or the angle is too large to cause no suitable suction contact. At the same time, a large compression preload is usually required, which is not convenient for flexible hand operation. Deimel et al. [43] designed a soft bionic hand completely imitating the structure of the human hand. It uses wire winding to divide the finger area so that the bending deformation of the outer side is greater than that of the inner side so as to achieve a shape similar to the bending of fingers. At the same time, it designs the palm structure opposite to the four fingers, which further simulates the posture of the human hand when grasping objects. This kind of driving mode is easy to realize, and the control is relatively simple. It can transmit the driving force from a long distance and ensure the small moment of inertia of the manipulator. However, there are also some problems. Generally, motors and transmission mechanisms are required, and the system is relatively bulky.

The structure of this paper is arranged as follows. In the first section, the overall modular design and the working principle of the gripper when facing different target objects will be described in detail. For different working conditions, the initial position of the gripper and the shape of the soft pneumatic grid are designed. In the second section, the mathematical theoretical model of bending deformation of soft gripper is established based on Yeoh constitutive model and classical differential geometry. In the third section, according to the existing research results, three kinds of gripper bending deformation models with different force cavity structures are designed for analysis and comparison, and the software clamping structure with the most suitable bending deformation is selected. The maximum input air pressure of the software gripper is obtained through the simulation experiment. The bending deformation of the gripper is analyzed, and the clamping capacity of the gripper is determined combined with the bending theoretical model. It also verifies the effectiveness of the simulation and the correctness of the mathematical theoretical model of bending deformation. Finally, the fourth section summarizes this paper.

## 2. Structural Design of Soft Gripper

The purpose of this paper is to design a general software gripper based on fluid drive. The main working object is small or complex objects. Therefore, the software gripper designed in this study needs to meet the following characteristics. The overall structure of the software manipulator needs to be compact and there is no redundant deformation in the whole clamping process so as to ensure that the deformation degree of the software material does not affect the accurate clamping when clamping small objects. The bending range is large, and the envelope clamping can be carried out in a reasonable attitude when dealing with complex surface objects. The driving mode is simple, and there is no additional energy except air.

**Figure 1:**
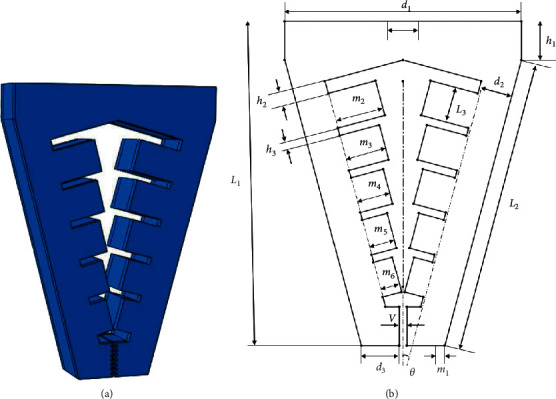
Structure of soft manipulator: (a) three-dimensional model of soft manipulator; (b) specific dimensions parameters of soft manipulator.

Based on the above design features, a soft gripper structure with two symmetrical grippers is proposed in this paper. Two working states are set for target objects of different sizes. When clamping a target object with a very small size, due to the expansion characteristics of soft material under pressure, the part in contact with the target object will also have a certain expansion deformation when bending, so it is difficult to clamp the target object accurately. In this case, an initial spacing of 1 mm was set in the opposite part of the fingertips of the soft gripper. When clamping small objects with a diameter of less than 1 mm, negative pressure can be applied to the soft gripper, the internal air cavity shrinks and deforms, and the soft gripper is driven to bend inward for clamping. Different from the current general working mode of the software gripper, which expands and bends the clamping claw by inputting positive pressure to envelop the target object, due to the small initial spacing, the software actuator only needs very little bending deformation, and the required driving air pressure is also reduced sharply. The deformation of the surface part of the software gripper in contact with the target object caused by the compression of the internal air cavity is almost negligible and no longer has a negative impact on clamping. When the target object is large in size or complex in shape, the second working mode can be adopted to ensure the universality of the software holder. Input positive pressure to expand and deform the clamping claw, when it is bent to an appropriate angle to properly envelope the target object, then apply negative pressure to shrink the soft clamping claw, and fit the surface of the object to achieve the purpose of clamping. When this working mode is adopted, the angle change of the soft holder with the increase of air pressure needs to be analyzed in detail to reasonably set the pneumatic grid structure. In order to deal with the target object with a small size, the initial distance between the gripper terminals has been set to be only 1 mm. In this paper, an inclination angle of about 15° is set for the soft gripper. When the air pressure is not input, the surface distance between the roots of the two soft grippers reaches 18 mm, which ensures that, in the process of compression, bending, and deformation of the soft gripper, the root position can extend a wider distance and a wider range of space can be selected when enveloping the target object. At the same time, the internal aerodynamic grid is improved, and the special design of decreasing thickness is adopted. The deformation degree of each segment of the pneumatic mesh decreases step by step, the curvature of each segment of the soft gripper is adjusted, and the clamping performance is improved by increasing the contact area with the target object. The three-dimensional structure of the software holder is shown in Figure 1, and the structural dimensions are shown in Table 1.

## 3. Theoretical Modeling of the Actuator

Different from ordinary materials, rubber materials are a collection of viscoelasticity and hyperelasticity. It mainly includes elastic statistical theory [44], finite strain elastic theory [45], and strain energy function form [46]. When we define material properties in ABAQUS software, we need to analyze them according to specific constitutive model and experimental data.

**Assumption 1 .**

In finite element analysis, the material is regarded as isotropic and characterized by strain energy per unit volume. It is regarded as incompressible material, which means that the total volume remains unchanged throughout the deformation period.

According to the theory of continuum mechanics [47], the deformation tensor of each volume unit is expressed as

(1) $$\begin{array}{c} W=\overset{N}{\underset{i+j=1}{\sum }}C_{ij}{\left(l_{1}-3\right)}^{i}{\left(l_{2}-3\right)}^{j}+\overset{N}{\underset{k=1}{\sum }}\frac{1}{D_{k}}{\left(l_{3}^{2}-1\right)}^{2k}. \end{array}$$

In the above equation, *W* is the strain energy density function of rubber material, *C* and *D* are relationship constants, and *l* and *λ* are the deformation tensor and principal elongation of the material.

The relationship between deformation tensor *l* and principal elongation *λ* is as follows:

(2) $$\begin{array}{c} l_{1}=\lambda_{1}^{2}+\lambda_{2}^{2}+\lambda_{3}^{2},\\ l_{2}={\left(\lambda_{1}\lambda_{2}\right)}^{2}+{\left(\lambda_{2}\lambda_{3}\right)}^{2}+{\left(\lambda_{1}\lambda_{3}\right)}^{2},\\ l_{3}={\left(\lambda_{1}\lambda_{2}\lambda_{3}\right)}^{2}. \end{array}$$

For rubber materials, since the total volume before and after deformation remains unchanged, let *l*_3_ in the above formula be equal to 1, which will not affect the energy transformation.

According to the above formula,

(3) $$\begin{array}{c} l_{1}=\lambda_{12}+\lambda_{22}+\lambda_{32},\\ l_{2}=\frac{1}{\lambda_{1}}+\frac{1}{\lambda_{2}}+\frac{1}{\lambda_{3}}. \end{array}$$

By substituting the deformation tensor expression [28], it can be simplified to

(4) $$\begin{array}{c} W=\overset{N}{\underset{i+j=1}{\sum }}C_{ij}{\left(l_{1}-3\right)}^{i}{\left(l_{2}-3\right)}^{j}. \end{array}$$

When the value of strain potential energy order *N* is 3, according to the above formula, the expression of the Yeoh constitutive model is

(5) $$\begin{array}{c} W=C_{10}\left(l_{1}-3\right)+C_{20}{\left(l_{1}-3\right)}^{2}+C_{30}{\left(l_{1}-3\right)}^{3}. \end{array}$$

In the Yeoh constitutive model, the principal stress of material *f* has

(6) $$\begin{array}{c} f_{1}=2\left(\lambda_{1}^{2}-\frac{1}{\lambda }\right)\left[C_{10}+2C_{20}\left(l_{1}-3\right)+3C_{30}{\left(l_{1}-3\right)}^{2}\right]. \end{array}$$

For the material of the soft gripper, the silicone material e620#a with greater flexibility is selected by referring to the existing literature [48]. The material has the advantages of good flexibility and large flexibility and meets the requirements of the software drive of the gripper.

Through experiments, the principal stress of rubber material at several groups of specific elongation is measured to calculate the Yeoh model constant of rubber material. The experimental simulation can be carried out by importing ABAQUS.

**Figure 2:**
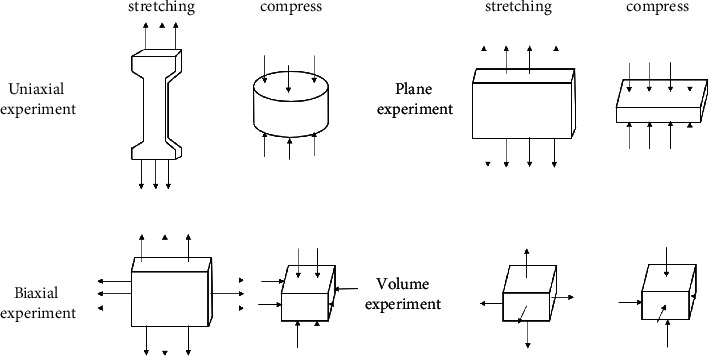
Characteristic experiment of rubber material.

The stress-strain characteristic parameters of rubber materials need to be obtained through a series of experiments. At present, the main rubber material characteristic experiments mainly include the following eight categories: uniaxial tensile test, uniaxial compression test, biaxial tensile test, biaxial compression test, plane tensile test, plane compression test (pure shear), volume tensile test, and volume compression test, as shown in Figure 2.

In this study, the static mechanical properties of rubber materials were tested according to the Chinese national recommended standard GB/T 528-2009 [49].

**Figure 3:**
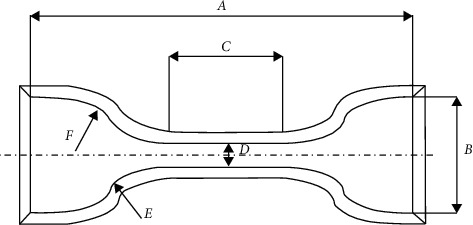
Shape and size of cutter.

Dumbbell-shaped specimen was selected as the tensile test specimen of this study to measure the material tensile rate. As shown in Figure 3 and Table 2, the 1A dumbbell-shaped sample selected in this experiment uses the size of the cutting knife specified in the standard.

The total length dimension A can be adjusted according to the actual situation, and the distance from the narrow middle section to both ends can be adjusted appropriately to ensure that the narrow part of the sample will not touch the tensile test device, prevent the phenomenon of “shoulder fracture” and ensure that tensile fracture will only occur in the test part in the middle.

**Figure 4:**
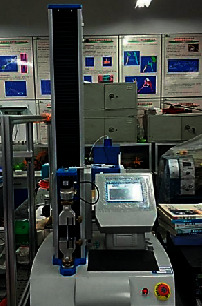
Tensile testing machine.

**Figure 5:**
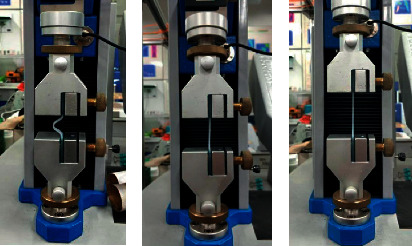
Experimental process of tensile testing machine.

As shown in Figure 4, HD-B609B-S electrohydraulic servo tensile tester is used in this study, which is an electronic tensile compressive material testing machine. Firstly, the two ends of the experimental rubber sample are clamped and fixed, in which the upper end is fixed with the force sensor and clamped with a clamp, and the other end is fixed by a clamp installed on the mobile platform. Turn on the motor to drive the ball screw to rotate so as to drive the connected platform to move at a uniform speed in the opposite direction. The rubber sample continues to elongate along both ends until the sample breaks. At this time, the force sensor will generate force signals, and the displacement sensor fixed on the mobile platform will also generate corresponding displacement signals. These signals are calculated to obtain the relationship curve between stress and strain. The experimental process is shown in Figure 5. Record the tensile value of the whole rubber sample in the uniform tensile process and the deformation of the rubber test.

**Figure 6:**
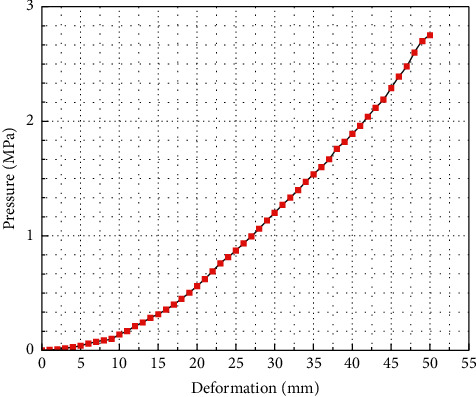
Relation curve between pressure and deformation of rubber sample in tensile test.

The deformation of the rubber sample is shown in Figure 6. Further process the averaged data, import it into Matlab, fit the obtained scatter diagram with a linear function, and solve the parameters in the Yeoh constitutive model: *C*_10_ = 0.08 MPa; *C*_20_ = 0.015 MPa.

According to the Yeoh model, the strain energy density function model *G* can be expressed in the form of binomial parameters:

(7) $$\begin{array}{c} W=C_{1}\left(l_{1}-3\right)+C_{2}{\left(l_{1}-3\right)}^{2}, \end{array}$$

where *C*_1_ and *C*_2_ are material constants.

Substituting equation (7) into equation (6), we can obtain

(8) $$\begin{array}{c} W=C_{1}\left(\lambda_{1}^{2}+\frac{1}{\lambda_{1}^{2}}-2\right)+C_{2}{\left(\lambda_{1}^{2}+\frac{1}{\lambda_{1}^{2}}-2\right)}^{2}. \end{array}$$

By deriving the strain energy density function *G* from the corresponding principal elongation ratio in the direction, the stress in each direction after the inner cavity is inflated can be deduced:

(9) $$\begin{array}{c} \sigma_{i}=\frac{\partial W}{\partial \lambda_{i}}. \end{array}$$

**Figure 7:**
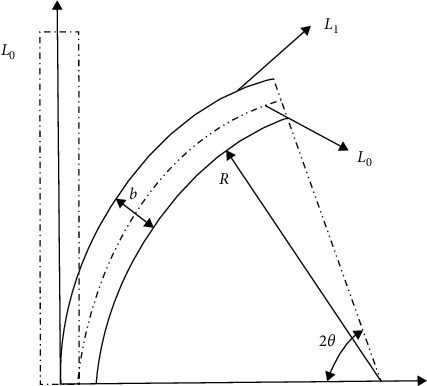
Bending deformation size.

Because the thickness and internal volume of each air chamber of the soft gripper designed in this paper are different, it cannot be simply regarded as a continuous body constant curvature bending model. It is difficult to accurately simulate the actual bending deformation with the current general mathematical model, so this paper proposes a mathematical model to solve it in segments. The whole software gripper is divided into five sections to establish and analyze the mathematical model. As shown in Figure 7, make the length of the single section model before deformation *L*_0_ and the length of the outer arc after deformation *L*_1_, the bending angle is *θ*, and the principal elongation ratio in the bending direction is

(10) $$\begin{array}{c} \lambda_{1}=\frac{L_{1}+2t_{2}\theta }{L_{1}}. \end{array}$$

In the above formula, *t* is the wall thickness of the air chamber. Substituting equations (1), (4), (8), and (10) into (9), it can be deduced that the relationship between elastic strain energy and stress in the direction of axial elongation is

(11) $$\begin{array}{c} \sigma_{1}=\frac{2}{\lambda_{1}}\left(\lambda_{1}^{2}-\frac{1}{\lambda_{1}^{2}\lambda_{1}^{2}}\right)\left(\frac{\partial G}{\partial l_{1}}+\lambda_{2}^{2}\frac{\partial G}{\partial l_{2}}\right). \end{array}$$

Since rubber materials are generally considered to be incompressible, the value of *l*_3_ is 1, which can be obtained by substituting into equation (3):

(12) $$\begin{array}{c} \lambda_{2}=\frac{1}{\lambda_{1}}. \end{array}$$

Substituting equations (10) and (12) into (11),

(13) $$\begin{array}{c} \sigma_{1}=\frac{8t_{2}\theta \left(L_{1}+t_{2}\theta \right)}{L_{1}\left(L_{1}+2t_{2}\theta \right)}\left[\frac{\partial G}{\partial l_{1}}+\frac{L_{1}}{{\left(L_{1}+2t_{2}\theta \right)}^{2}}\frac{\partial G}{\partial l_{2}}\right]. \end{array}$$

When the gas is filled into the gas chamber, the software holder is stressed and deformed. Take the single throttle chamber as an independent unit for analysis, which can be regarded as a confined space with balanced stress, and the stress on the inner wall of the gas chamber is the same. As shown in the figure, let the air pressure inside the air chamber be *p*, the thickness of the hollow air chamber be *m*, and the simplified model thickness of the single section soft holder be *b*.

**Assumption 2 .**

The pressure *p* in each inner cavity is evenly distributed, the input gas is an ideal gas, which can be approximated to isothermal conditions, and all orifices experience blocked flow. In addition, compared with the mechanical work of gas, the energy related to gas flow and heat transfer is considered to be negligible.

Through the mechanical analysis of the inflation process, the base section stress *σ*_2_ can be deduced from the moment balance generated during bending:

(14) $$\begin{array}{c} \sigma_{2}=\frac{p{\left(m-2b\right)}^{2}}{4b^{2}}. \end{array}$$

Substituting equations (12) and (14) into (8), we can obtain the relationship between the pressure *p*, the hollow cavity thickness *m*_*i*_, and the principal elongation ratio *λ*_1_ in a single section:

(15) $$\begin{array}{c} \lambda_{1}=\frac{p{\left(m_{i}-2b\right)}^{2}}{16C_{1}d^{2}}+1. \end{array}$$

Substituting equation (15) into (14), we can obtain the relationship between the pressure *p*, the hollow cavity thickness *m*, and the bending angle *θ*_*i*_ in a single section:

(16) $$\begin{array}{c} \theta_{i}=\frac{pL_{1}{\left(m_{i}-2b\right)}^{2}}{32tC_{1}d^{2}}. \end{array}$$

If the thickness of the inner air cavity of each section of the soft holder is *m*_1_ to *m*_5_, respectively, then the overall bending deformation can be obtained by accumulating the bending deformation results of each single section. The relationship between the pressure p, the hollow cavity thickness m, and the bending angle *θ* can be obtained in a single section:

(17) $$\begin{array}{c} \theta =\overset{5}{\underset{i=1}{\sum }}\frac{pL_{1}{\left(m_{i}-2b\right)}^{2}}{32tC_{1}d^{2}}. \end{array}$$

The classical differential geometry theory [50] parameterizes the space curve through its arc length *s*. The unit tangent vector *T*, the unit normal vector N, and the unit subnormal vector B are called the Flanner coordinate system. In vector calculus, Flanner's formula is used to describe the motion of particles in Euclidean space on a continuous differentiable curve. Flanner's formula describes the relationship among the tangent, normal, and subnormal directions of the curve.

**Figure 8:**
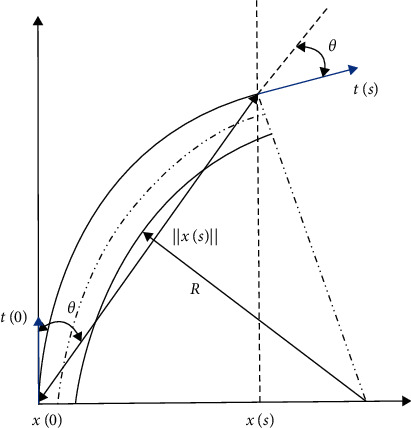
Angle and position vector during bending deformation.

As shown in Figure 8, the position vector *x* is defined as *x*=[*x*, *y*, *z*]^*T*^. Define the unit tangent vector of the space curve at *x* as *t*=*dx*/*ds*. Firstly, the unit normal vector *n* is defined so that *t·n*=0; then the unit subnormal vector *b* is defined so that *b*=*t* × *n*. Set the unit tangent vector set of the above principal normal, secondary normal, and spatial curve at *x* as a new spatial reference system. Set the scalar parameters *к* and *τ*, they represent the curvature and torque of the unit cell, respectively, and their values can be positive or negative. Then the three vector formulas can be written as

(18) $$\begin{array}{c} \frac{dt}{ds}=кn, \end{array}$$

(19) $$\begin{array}{c} \frac{dn}{ds}=-кt+\tau b\text{ ,} \end{array}$$

(20) $$\begin{array}{c} \frac{db}{ds}=-\tau n\text{ .} \end{array}$$

An important note is that equation (18) defines *τ* uniquely, but only *к*^2^ not *к* can be defined uniquely.

For the single section deformation of the pneumatic gripper, after simplified modeling, the curvature and torque of the simplified bending model can be constructed as a general function of the arc length in the curve. It can be seen from the simulation results that although the volume of the protruding inner cavity of each section is different, when analyzing the deformation of a single section, it can be regarded as a mathematical model with constant curvature bending and basically zero torque. Therefore, for the air cavity bending model of single section gripper, based on the assumption of constant curvature bending and zero torque, equation (18) can be written as the following linear differential equation:

(21) $$\begin{array}{c} t^{'\prime }+{к}^{2}t=0\text{ .} \end{array}$$

*t*′ represents the differential of the unit tangent vector *t* in the arc length *s* direction. When the initial conditions *t*_0_=*t*(0) and *n*_0_=*n*(0) are substituted into equation (21), the tangent vector equation in the *s* direction can be written as

(22) $$\begin{array}{c} t\left(s\right)=t_{0}\cos\left(кs\right)+n_{0}\sin\left(кs\right). \end{array}$$

The position vector can be obtained by integrating equation (22) from 0 to s:

(23) $$\begin{array}{c} x\left(s\right)=\frac{t_{0}}{к}\sin\left(кs\right)+\frac{n_{0}}{к}\left\{1-\cos\left(кs\right)\right\}. \end{array}$$

Combined with equation (23), using the right-hand rule, rotate 90 degrees around the unit pair normal vector *b* to obtain the vector *n*. Define *t*_0_=[*t*_0*x*_，*t*_0*y*_]^*T*^; then *n*_0_ can be expressed as *n*_0_=[−*t*_0*y*_，*t*_0*x*_]^*T*^. Substitute *t*_0_ and *n*_0_ into equation (23):

(24) $$\begin{array}{c} {\left[x,y\right]}^{T}=\frac{1}{к}{\left[t_{0x},t_{0y}\right]}^{T}\sin\left(кs\right)+\frac{1}{к}{\left[-t_{0y}，t_{0x}\right]}^{T}\left\{1-\cos\left(кs\right)\right\}. \end{array}$$

The modulus of the vector *x* is

(25) $$\begin{array}{c} \left\|x\right\|=\sqrt{x^{2}+y^{2}}, \end{array}$$

(26) $$\begin{array}{c} x^{2}=\frac{1}{{к}^{2}}t_{0x}^{2}\sin^{2}\left(кs\right)-\frac{2}{{к}^{2}}t_{0x}t_{0y}\sin\left(к\;s\right)\left\{1-\cos\left(кs\right)\right\}, \end{array}$$

(27) $$\begin{array}{c} y^{2}=\frac{1}{{к}^{2}}t_{0y}^{2}\sin^{2}\left(кs\right)+\frac{2}{{к}^{2}}t_{0x}t_{0y}\sin\left(кs\right)\left\{1-\cos\left(кs\right)\right\},\\ +\frac{1}{{к}^{2}}t_{0x}^{2}\left\{1-2\;\cos\left(кs\right)+\;\cos^{2}\left(кs\right)\right\}. \end{array}$$

Define an initial unit tangent vector *t*_0_ whose modulus is 1, ‖*t*_0_‖=1. Substituting equations (26) and (27) into (25), we can deduce

(28) $$\begin{array}{c} \left\|x\right\|=\frac{\sqrt{2}}{к}\sqrt{1-\cos\left(кs\right)}. \end{array}$$

As shown in the figure, let the angle between vector *x* and vector *t*_0_ be *θ*, and it can be deduced that

(29) $$\begin{array}{c} \sin\left(\theta \right)=\frac{\left\|x\times t_{0}\right\|}{\left\|x\right\|\left\|t_{0}\right\|} \end{array}$$

(30) $$\begin{array}{c} \cos\left(\theta \right)=\frac{x\cdot t_{0}}{\left\|x\right\|\left\|t_{0}\right\|}=\frac{\sqrt{2}}{2}\frac{\sin\left(кs\right)}{\sqrt{1-\cos\left(кs\right)}}. \end{array}$$

Combining equations (29) and (30), the included angle can be derived by using triangular identity *θ* expression for

(31) $$\begin{array}{c} \theta =\frac{кs}{2}. \end{array}$$

Substituting equations (31) into (28), we can obtain that

(32) $$\begin{array}{c} \left\|x\right\|=\frac{s}{\theta }\sin\left(\theta \right). \end{array}$$

When the bending conditions of the five deformed gas chambers are superimposed, the mode of the position vector at the tip of the gripper is

(33) $$\begin{array}{c} \left\|x\right\|=\overset{5}{\underset{i=1}{\sum }}\frac{s}{\theta_{i}}\sin\left(\theta_{i}\right). \end{array}$$

Equation (33) is a function of angle, which couples the size of position vector *x* and the relationship of angle. When the section of the simplified model of the soft gripper is bent, it can basically be equivalent to a connecting rod structure with variable length through the above equation. After the included angle *θ* and the expression of modulus *x* are obtained, the expression of the terminal position vector *x* of the software gripper can be derived by combining equations (10), (16), and (23):

(34) $$\begin{array}{c} x=\frac{1}{t_{0}}\cdot \overset{5}{\underset{i=1}{\sum }}\frac{s}{\theta_{i}}\sin\left(\theta_{i}\right)\cdot \;\cos\overset{5}{\underset{i=1}{\sum }}\frac{pL_{1}{\left(m_{i}-2b\right)}^{2}}{32tC_{1}d^{2}}. \end{array}$$

## 4. Experimental Simulation and Data Analysis

In the part of the previous section, the mathematical model of the bending deformation of each soft air cavity has been derived by subsection processing. Through the simulation analysis from the mathematical angle, the relationship between the bending angle, elongation ratio, position vector, and the parameters such as driving air pressure and structural size is obtained. However, different from the traditional rigid mechanical structure, it is still impossible to establish a fully accurate mathematical model for the bending deformation caused by hyperelastic materials. With the change of the working environment or the physical parameters of the target object, the analysis results of the mathematical model will still have a certain error with the actual operation. In order to further simulate and analyze the motion mode of the soft-body gripper, obtain the influence of various physical parameters on the stress and strain of the soft-body gripper, and predict the output force when envelope clamping with the target object, this section will analyze the deformation of the general pneumatic soft-body gripper under different air pressures based on ABAQUS simulation platform.

**Figure 9:**
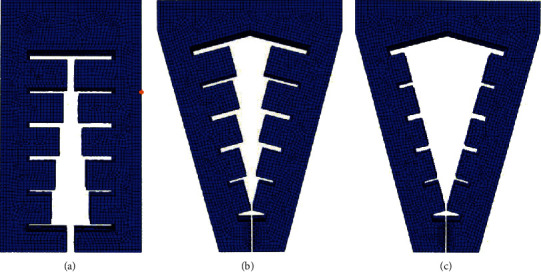
Shape of soft gripper with three different shapes driving air cavity before deformation. (a) Type I. (b) Type II. (c) Type III.

The structure of the soft gripper designed in this study is relatively special. The difference from the current general soft mechanical structure is that the protruding internal air cavity not only plays the role of bending and deformation of the gripper driven by positive pressure expansion but also envelops or clamps the object when subjected to negative pressure, giving a certain force to the target object. For small objects with a diameter of no more than 5 mm, the horizontally opposite part of the final end can be used for direct clamping. However, when facing large objects, in order to ensure the clamping efficiency, the bending characteristics of the soft air cavity can be used to shrink after being subjected to negative pressure through envelope, and the protruding air cavity at the upper end can be used for contact to provide clamping capacity. If the whole soft gripper model makes constant curvature bending movement when pressed, the curvature of the terminal part of the gripper is the same as that of the upper part. If the surface curvature of the object is greater than that of the actuator, extrusion may occur and affect the clamping efficiency. For such cases, Glick et al. [15] improved an air-driven soft actuator to generate bending deformation with nonconstant curvature by gradually reducing the thickness of the air cavity inside the soft gripper from the root to the tip. In this way, with the increase of the driving air pressure, the two opposite software actuators can surround the surface of the target object with a better envelope angle. On the basis of this work, this study further improves the above work and designs a software gripper with a certain inclination angle between the two actuators. In addition to changing the thickness of each internal cavity, it also adjusts the distance between each section relative to the top of the cavity. In order to verify the influence of the above conditions on the curvature change, three types of finger actuators are designed and imported into the ABAQUS platform, and the material parameters *C*_10_ = 0.08 MPa and *C*_20_ = 0.015 MPa are set in the software. Add fixed constraints and input air pressure to simulate the positive pressure and negative pressure of the software holder. Set the constraint mode as binding constraint, and take the top of the software holder as the fixed end, so that the input air pressure is evenly distributed on the inner surface of the whole force chamber. Simulation and comparative analysis are carried out under the same air pressure input conditions. The shape of the soft gripper with three different shapes driving the air cavity before deformation is shown in Figure 9.

**Figure 10:**
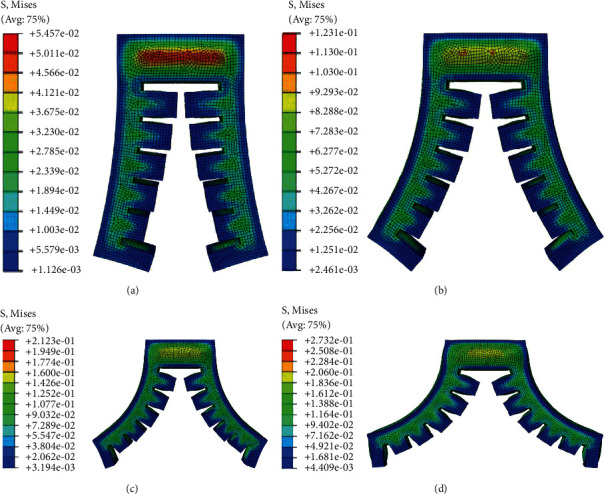
Bending deformation of type I soft gripper under four experimental air pressures: (a) the value of the input air pressure is 0.008 MPa; (b) the value of the input air pressure is 0.014 MPa; (c) the value of the input air pressure is 0.020 MPa; (d) the value of the input air pressure is 0.025 MPa.

**Figure 11:**
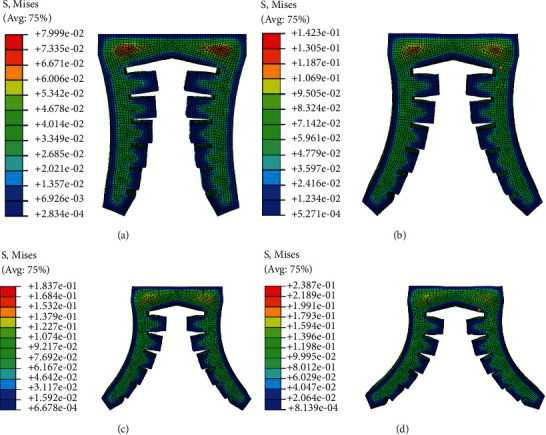
Bending deformation of type II soft gripper under four experimental air pressures: (a) the value of the input air pressure is 0.008 MPa; (b) the value of the input air pressure is 0.014 MPa; (c) the value of the input air pressure is 0.020 MPa; (d) the value of the input air pressure is 0.025 MPa.

**Figure 12:**
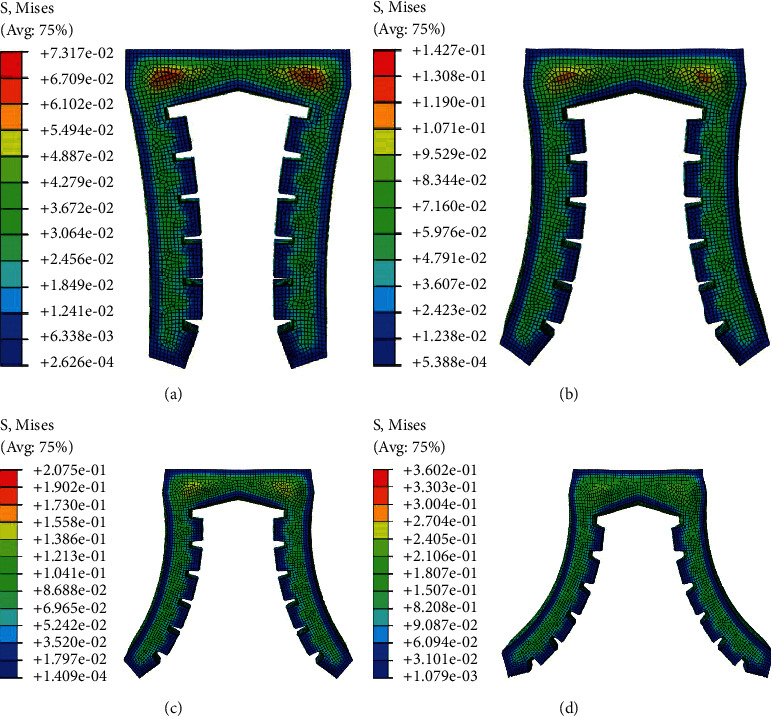
Bending deformation of type III soft gripper under four experimental air pressures: (a) the value of the input air pressure is 0.008 MPa; (b) the value of the input air pressure is 0.014 MPa; (c) the value of the input air pressure is 0.020 MPa; (d) the value of the input air pressure is 0.025 MPa.

When the soft actuator is opened under positive pressure, the maximum diameter and weight of the target object depend on the maximum pressure it can bear and the maximum deformation. In order to obtain the limit air pressure and the maximum deformation angle, the deformation simulation of the gripper under different positive pressures is carried out. In order to better simulate and analyze the bending deformation of each actuator structure and compare it, four groups of experimental input air pressures are set. When the input air pressure *p* is 0.008 MPa, 0.014 MPa, 0.20 MPa, and 0.25 MPa, respectively, the deformation of each soft actuator is shown in Figures 10–12, respectively.

**Figure 13:**
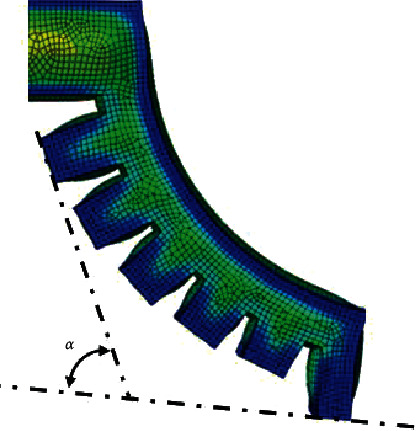
Included angle *α* between the tangent of the root cavity surface and the tangent of the tip cavity surface.

**Figure 14:**
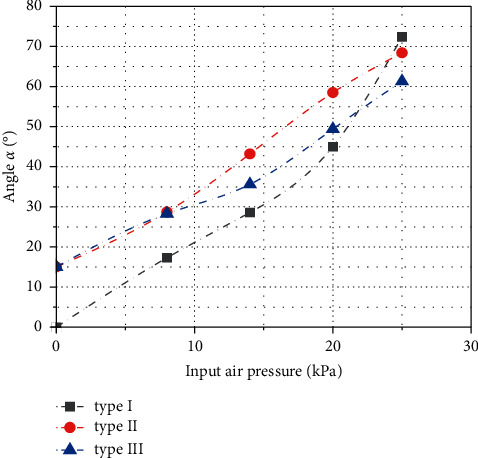
Relationship curve between the included angle *α* of three kinds of soft grippers and the change of input air pressure.

Through image processing and numerical analysis, the bending deformation of the three soft actuators under positive pressure is quantitatively compared. As the main working form is to expand under pressure, open the soft actuator to envelop the target object as much as possible, then apply negative pressure contraction, and the sidewall squeezes the target object to give force for clamping, so when the surface of the target object is with complex shape, the bending mode of the soft actuator must be as smooth as possible in order to ensure that there are more inner surfaces to bind and fit with the target object as much as possible. Measure the included angle *α* between the surface tangent of the root cavity and the surface tangent of the tip cavity of each type of soft actuator under each experimental pressure. As shown in Figures 13 and 14, the initial included angle *α* of gripper type I is 0. With the gradual increase of input air pressure, when the input air pressure reaches 25 kPa, the included angle *α* between the tangents of the two surfaces reaches 72.4°. The initial included angle of gripper type II and gripper type III is 15°, when the input air pressure reaches 25 kPa; due to the difference in the shape of the internal aerodynamic grid, the angle between the surface tangent of the root cavity and the surface tangent of the tip cavity of the gripper type II increased 68.4°, and the growth curve is relatively smooth, which can be approximately fitted by an equation with a slope of 2.55. The included angle between the tangents of the two surfaces of the gripper type III increased to 61.3°. The growth trend is slow; the variation range of the included angle is slightly smaller than that of gripper type II.

**Figure 15:**
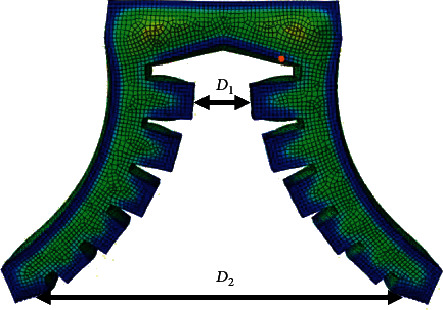
Surface spacing of air cavity of soft gripper.

**Figure 16:**
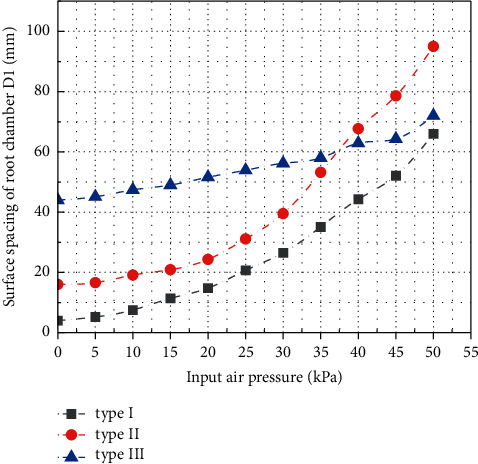
Relationship curve of surface spacing *D*_1_ of air cavity at the root of three kinds of soft grippers with input air pressure.

**Figure 17:**
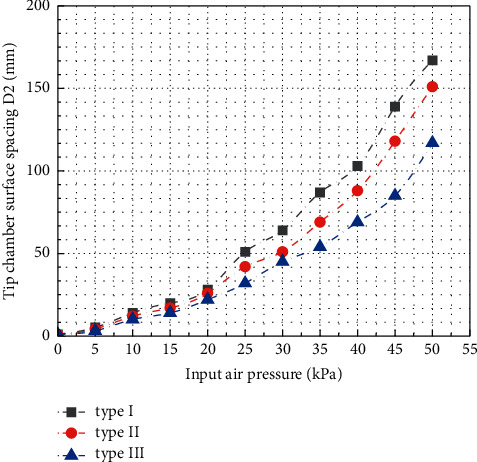
Relationship curve of surface spacing *D*_2_ of air cavity at the root of three kinds of soft grippers with input air pressure.

The surface spacing *D*_1_ of the air cavity at the root of the three types of soft grippers is shown in Figure 15. It can be seen that, due to structural differences, at the initial position, the surface spacing *D*_1_ of the root air cavity of type III gripper is 44 mm, which is much larger than that of type I and type II. With the gradual increase of the input air pressure, when the input air pressure reaches about 37 kPa, the surface spacing *D*_1_ increases to about 61 mm, which is the same as the surface spacing of the root air cavity of the gripper type II. When the input air pressure reaches 50 kPa, the surface spacing *D*_1_ increases to 72.8 mm, and the displacement increment of the root is about 28 mm. The surface spacing *D*_1_ of the root air cavity at the initial position of type II gripper is 16 mm. When the input air pressure reaches 50 kPa, *D*_1_ increases to 94.3 mm. The surface spacing *D*_1_ of the root air chamber at the initial position of type I gripper is only 4 mm, but when the input air pressure reaches 50 kPa, the surface spacing *D*_1_ increases to 66.5 mm, as shown in Figure 16. Through analysis and comparison, it can be found that the shape of compressed air cavity of type I and II soft grippers is the same; with the increase of the input air pressure, the growth rate of the surface spacing *D*_1_ of the root air chamber is almost the same. However, due to the low initial spacing of the root cavity surface of type I gripper, the final simulation result is still lower than that of type II. This shows that the opening and closing of the root of the soft gripper are not only related to the shape of the compressed air cavity but also greatly affected by the distance of the initial position. The surface spacing *D*_2_ of the tip cavity is shown in Figure 17. In the initial position, the surface spacing *D*_2_ of the tip air cavity of the gripper type I, the gripper type II, and the gripper type III is 1 mm. As the input air pressure increases gradually, the growth curve of surface spacing *D*_2_ can be fitted by the third-order polynomial. When the input air pressure gradually increases to 50 kPa, the surface spacing *D*_2_ of the tip air cavity of the three grippers is 167.8 mm, 151.0 mm, and 117.7 mm, respectively. The surface spacing of the tip cavity also represents the expansion and bending of the soft actuator under pressure to a certain extent. For the soft gripper, the greater the distance between the surface of the air cavity at the root and tip, the more intense the deformation when opening; it usually means that the target object that can adapt to a wider range of size changes will also affect the clamping efficiency to a certain extent.

**Figure 18:**
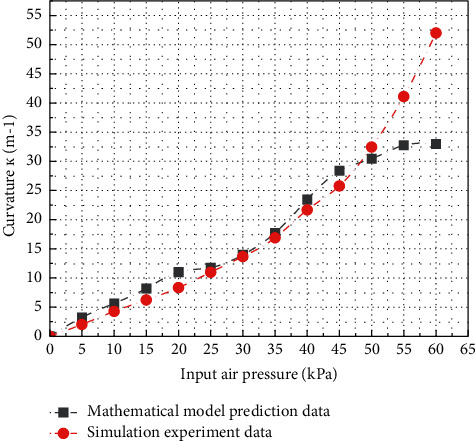
Curve of curvature of soft gripper varying with input pressure and prediction data.

Based on the above data processing and the comparison of advantages and disadvantages, the software gripper type II is finally selected for the next experimental analysis. When clamping an object with large overall size or complex surface shape, the envelope degree of the software gripper to the target object determines the success rate of clamping. Therefore, the software gripper is simulated under positive pressure to analyze the bending deformation under positive pressure. With the increase of the input air pressure, the curvature of the software gripper is shown in Figure 18. Combined with the derivation of the mathematical model in the previous section, the simulation experimental data are compared with the mathematical prediction results. It can be seen that before the input air pressure gradually increases from 0 to 45 kPa, there is little difference between the curvature simulation experimental data of the soft gripper and the mathematical prediction results, and the maximum error is no more than 3.5%. However, after the input air pressure reaches 50 kPa, the deformation degree of the software holder reaches the bottleneck in the simulation analysis, and the growth rate decreases rapidly. When the input air pressure increases from 50 kPa to 55 kPa, the curvature changes by only 7.5%, and when the input air pressure increases from 55 kPa to 60 kPa, the curvature changes by only 0.61%, which is very different from the prediction results of the mathematical model. Through the above experimental data, the theoretical correctness of the bending deformation model of the soft gripper is proved. When the input air pressure does not exceed 46 kPa, the compression and expansion deformation of the soft gripper is basically stable and in line with the model. The limit air pressure of the soft gripper is about 50 kPa. When it exceeds 50 kPa, the nonideal deformation of the soft gripper increases significantly. At this time, it can be basically regarded as the limit bending deformation degree of the soft gripper.

**Figure 19:**
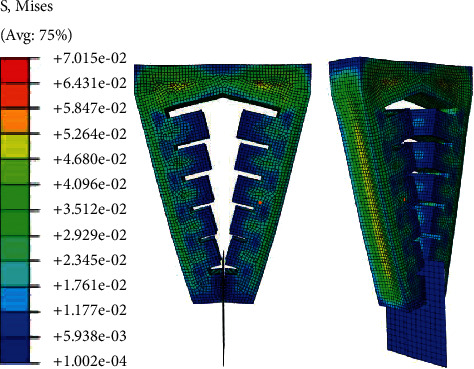
Negative pressure simulation of soft gripper.

**Figure 20:**
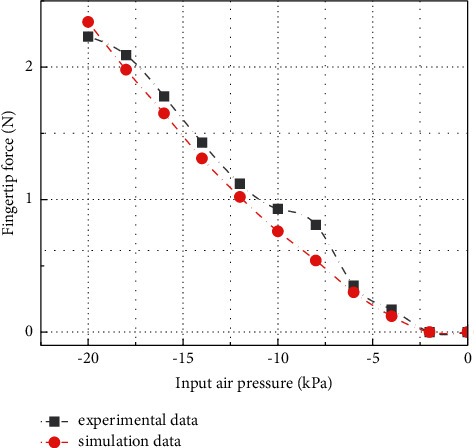
Relationship curve of gripper curvature with input pressure and prediction data.

When clamping an object with small shape and size, the main working form is to rely on the convex part at the tip for fixed clamping. As shown in Figure 19, the negative pressure simulation of the software holder is carried out. In order to determine the clamping efficiency when clamping small objects, this study simulates the case of a soft gripper clamping an iron sheet with a thickness of only 0.1 mm and uses a force sensor to extract the force. The experimental results are shown in Figure 20.

## 5. Conclusions

A general pneumatic soft gripper is proposed in this paper. The bending deformation models of three different force-bearing cavity structures are analyzed and compared, and the soft gripper structure with the most suitable bending deformation is selected. Combined with the torque balance theory, the mathematical theoretical model of bending deformation of soft gripper is established based on Yeoh constitutive model and classical differential geometry, and the deformation of soft finger is analyzed. The correctness and rationality of the model are verified by the comparative analysis of the mathematical theoretical model and the simulation experimental results.

## Figures and Tables

**Table 1:** Dimensional parameters of software holder.

Parameters	Values
Root width *d*_1_ (mm)	92
Distance from bottom of air chamber to sidewall *d*_2_ (mm)	13
Terminal width *d*_3_ (mm)	14.7
Inclination angle *θ* (°)	15
Total length *L*_1_ (mm)	126
Length of inclined part *L*_2_ (mm)	115
Total width of single throttle chamber *L*_3_ (mm)	14
Top spacing of terminal air chamber *v* (mm)	1
Height of vertical part *h*_1_ (mm)	15
Section I chamber spacing *h*_2_ (mm)	5
Chamber spacing *h*_3_ (mm)	3
Overall wall thickness *m*_1_ (mm)	3
First throttle chamber depth *m*_2_ (mm)	19
Second throttle chamber depth *m*_3_ (mm)	16
Third throttle chamber depth *m*_4_ (mm)	13
Fourth throttle chamber depth *m*_5_ (mm)	10
Fifth throttle chamber depth *m*_6_ (mm)	7.5

**Table 2:** Cutter size of dumbbell type 1A specimen.

Sample type	1A
Total length (minimum) A(mm)	100
End width B (mm)	25.0 ± 1.0
Length of narrow part C (mm)	20.0 ± 2.0
Length of narrow part D (mm)	5.0 ± 0.1
Outer transition edge radius E (mm)	11.0 ± 1.0

## Data Availability

The data used to support the findings of this study are available from the corresponding author upon request.
